# Antibiotic Susceptibility Testing of Grown Blood Cultures by Combining Culture and Real-Time Polymerase Chain Reaction Is Rapid and Effective

**DOI:** 10.1371/journal.pone.0027689

**Published:** 2011-12-14

**Authors:** Judith Beuving, Annelies Verbon, Firza A. Gronthoud, Ellen E. Stobberingh, Petra F. G. Wolffs

**Affiliations:** Department of Medical Microbiology, Care And Public Health Research Institute (CAPHRI), Maastricht University Medical Center, Maastricht, The Netherlands; The Scripps Research Institute, United States of America

## Abstract

**Background:**

Early administration of appropriate antibiotic therapy in bacteraemia patients dramatically reduces mortality. A new method for RApid Molecular Antibiotic Susceptibility Testing (RAMAST) that can be applied directly to positive blood cultures was developed and evaluated.

**Methodology/Principal Findings:**

Growth curves and antibiotic susceptibility of blood culture isolates (*Staphylococcus aureus*, enterococci and (facultative) aerobic Gram-negative rods) were determined by incubating diluted blood cultures with and without antibiotics, followed by a quantitative universal 16S PCR to detect the presence or absence of growth. Testing 114 positive blood cultures, RAMAST showed an agreement with microbroth dilution of 96.7% for Gram-negative rods, with a minor error (false-susceptibility with a intermediate resistant strain) rate of 1.9%, a major error (false resistance) rate of 0.8% and a very major error (false susceptibility) rate of 0.6%. Agreement for *S.aureus* was 97.9%, with a very major error rate of 2.1%. *Enterococcus* species showed 95.0% agreement, with a major error rate of 5.0%. These agreements are comparable with those of the Phoenix system. Starting from a positive blood culture, the test was completed within 9 hours.

**Conclusions/Significance:**

This new rapid method for antibiotic susceptibility testing can potentially provide accurate results for most relevant bacteria commonly isolated from positive blood cultures in less time than routine methods.

## Introduction

Invasion of bacteria in the bloodstream (bacteraemia) can result in sepsis. Sepsis occurs in about two percent of all hospitalized patients and in as much as 37.4% of all ICU patients and the incidence is rising [1], [2], [3], [4]. Mortality is high, varying from 14 to 57% [5]. Although this percentage has declined during the past decades, due to the rising incidence of sepsis [1], the total number of deaths through sepsis is still rising, making it the tenth leading cause of death in the United States [6].

Early administration of appropriate antibiotics reduces mortality of sepsis dramatically [7], [8], [9]. Usually, empiric therapy, consisting of one or more broad spectrum antibiotics, is started as soon as bacteraemia is suspected. Broad spectrum antibiotics are active against most bacteria causing bacteraemia and are often used in combination. However, their use favors the selection of antibiotic resistant bacteria [10]. Moreover, the more antibiotics a patient uses, the greater the risk of drug toxicity. Additionally, the empiric antibiotic therapy may not cover the causative micro-organism, especially with the rising incidence of multi-drug resistant bacteria [10]. This is the case in up to 40% of all bacteraemia patients [9], [11], [12].

Rapid analysis of the antibiotic susceptibility pattern of the causative micro-organism in bacteraemia leads to earlier targeting of antibiotic therapy and may be lifesaving [13], [14], [15]. A major drawback of the conventional blood culture systems is that once blood cultures are positive, additional one or two overnight incubations are required for identification of the causative micro-organism, antibiotic susceptibility testing and targeting of antibiotic therapy.

A new method combining culture and real-time PCR for antibiotic susceptibility testing within 7 hours was reported by Rolain et al. [16]. In that study, reference strains were cultured for several hours in the presence of an antibiotic, after which growth was measured with real-time PCR. The absence or presence of growth indicated susceptibility or resistance to the tested antibiotic.

The aim of this study was to modify the method of Rolain et al. in order to develop a new rapid method for RApid Molecular Antibiotic Susceptibility Testing (RAMAST) which can be applied directly on positive blood cultures. The study was conducted in 2 phases: (I) establishment of growth curves of bacteria harvested from positive blood cultures using real-time PCR, to determine the required minimal incubation time for adequate antibiotic susceptibility testing, and (II) antibiotic susceptibility testing of 114 blood culture isolates with RAMAST, to investigate the accuracy of RAMAST in clinical isolates.

## Materials and Methods

### Ethics statement

All data in this study were analyzed anonymously. Therefore, no consent from the patients was required and the ethics committee did not have to be approached. This is in agreement with the code for proper use of human tissue as formulated by the Dutch Federation of Medical Scientific Societies and the policy of the Medical Ethics Committee of the Maastricht University Medical Center.

### Sample collection

The study was performed in the Department of Medical Microbiology of the Maastricht University Medical Center, a 750-bed referral hospital. Blood cultures were incubated in the Bactec automated blood culture device (Bactec™ 9240, BD Diagnostic Systems, Sparks, MD, USA). Bacterial growth in the bottles was detected through continuous monitoring of the CO_2_ level by the Bactec device. Between October 2009 and July 2010, all blood cultures that were signaled positive in the previous 24 hours and contained *Staphylococcus aureus*, *Enterococcus* species or (facultative) aerobe Gram-negative rods (GNRs) were included. Blood cultures with anaerobes, *Streptococcus* species, coagulase-negative staphylococci (CoNS) or with growth of more than one species were excluded from the study.

### Establishment of growth curves of bacteria harvested from positive blood cultures

Positive blood cultures (0.5 mL) were diluted 10^−5^ in double concentrated Mueller Hinton II broth (212322, BBL™ Mueller Hinton II Broth (Cation-Adjusted), BD Diagnostic Systems). 50 µl of this diluted blood culture was added to each well of a microtiter plate containing 50 µl of antimicrobial solution (table 1) or sterile demineralised water. The concentrations of the antibiotics were based on the breakpoints for susceptibility according to the CLSI-guideline for microbroth dilution [17]. This plate was incubated for 0, 2, 4, 6 and 8 hours at 37°C. After incubation, the content of the wells was transferred to sterile tubes. These were centrifuged at 16000× *g* for 5 minutes, after which the supernatant was removed. The remaining pellet of bacteria was suspended in demineralised water and stored at 4°C until PCR was performed.

**Table 1:** Tested antibiotics^a^ and concentrations^b^.

Gram-positive panel	Gram-negative panel
AMX 0.25 mg/l^S^	AMX 8 mg/l^Eb^
AMX 8 mg/l^Ec^	AMC 8/4 mg/l^Eb^
OXA 2 mg/l^S^	PIP 16 mg/l^Eb^^, ^ N
VAN 2 mg/l^S^	PIP 64 mg/l^P^
VAN 4 mg/l^Ec^	TZP 16/4 mg/l^Eb^^, ^ N
GEN 4 mg/l^S^	CIP 1 mg/l^Eb^^, ^ P ^, ^ N
STX 2/38 mg/l^S^	CAZ 1 mg/l^Eb^^, ^ N
	CAZ 8 mg/l^Eb^^, ^ P ^, ^ N
	GEN 4 mg/l^Eb^^, ^ P ^, ^ N
	STX 2/38 mg/l^Eb^^, ^ P ^, ^ N
Mixture of antibiotics^A^ containing: VAN 4 mg/l, PIP 64 mg/l, CIP 1 mg/l, GEN 4 mg/l	
Growth control (water)^A^	

a AMX, amoxicillin; OXA, oxacillin; VAN, vancomycin; GEN, gentamicin; STX, sulfamethoxazole/trimethoprim; AMC, amoxicillin/clavulanate; PIP, piperacillin; TZP, piperacillin/tazobactam; CIP, ciprofloxacin; CAZ, ceftazidime;

b These concentrations were based upon the CLSI breakpoints for susceptibility.

s For *Staphylococcus aureus*.

Ec For *Enterococcus* species.

Eb For Enterobacteriaceae.

P For *Pseudomonas aeruginosa*.

N For non-fermenters.

A For all strains.

The real-time PCR assay used in this study was described previously [18]. In short, the PCR-reaction mix included two universal 16S rRNA gene primers (5′-TGGAGAGTTTGATCCTGGCTCAG-3 and 5′-TACCGCGGCTGCTGGCAC-3′), iQ SYBR Green Supermix (Bio-Rad Laboratories BV, Veenendaal, the Netherlands), water and bacterial isolate. The MyiQ Single-Color Real-Time PCR Detection System (Bio-Rad Laboratories BV) was used for amplification and melt curve analysis. The threshold was calculated automatically.

The threshold cycle value (Ct-value) was plotted against time to establish a growth curve, in which a descending line indicates growth.

### Assessment of antibiotic susceptibility using RAMAST

After Gram-staining, 5 ml of grown blood culture was aspirated from the blood culture bottle and the aspirate was injected in a Serum Separator Tube (SST) (BD). This tube was centrifuged at 2000× *g* for 10 minutes, after which the supernatant was discarded. Bacteria were harvested from the gel layer using a sterile cotton swab and suspended in 0.9% saline until a 0.5 McFarland standard suspension was obtained. This suspension was diluted in double-concentrated Mueller-Hinton II broth to an inoculum of 5×10^5^ CFU/mL. This dilution was incubated in a microtiter plate with and without antibiotics (table 1) for 6 hours at 37°C.

After incubation, the content of each well was processed for PCR as described above, as well as a sample of diluted bacterial suspension that was not incubated at 37°C but had instead been stored at 4°C. To prevent inhibition of the PCR-reaction due to high loads of bacteria, all samples were diluted 10 times in demineralized water.

To determine antibiotic susceptibility, cut-off Ct-values were calculated. These cut-off Ct-values were chosen from initial growth curve experiments to obtain optimal agreement for each drug-organism combination (data not shown). They represent the mean of the Ct-value of the positive and negative growth control. The positive growth control was the sample that was incubated without antibiotics. For GNRs, the sample incubated with the antibiotic mixture (see table 1) was used as negative growth control. For *S. aureus* and *Enterococcus* spp. the unincubated sample was used as negative growth control.

To calculate the cut-off Ct-values, the following formula were used:

Cut-off Ct-value 1 = Ct-value positive growth control+0.5×(Ct-value negative growth control – Ct-value positive growth control)

And:

Cut-off Ct-value 2 = Ct-value positive growth control+0.25×(Ct-value negative growth control – Ct-value positive growth control)

For *S. aureus* and enterococci, the two cut-off values were used as follows:

Cut-off value 1: for vancomycin and gentamicin
Cut-off value 2: for amoxicillin, oxacillin and trimethoprim/sulfamethoxazole

For GNRs, the two cut-off values were used for the following antibiotics:

Cut-off value 1: for amoxicillin, amoxicillin/clavulanate, ciprofloxacin, gentamicin and trimethoprim/sulfamethoxazole
Cut-off value 2: for piperacillin, piperacillin/tazobactam and ceftazidim

A Ct-value higher than the cut-off Ct-value indicated susceptibility for the antibiotic. A Ct-value lower than the cut-off value indicated antibiotic resistance.

If a positive blood culture grew less than 1 log (a difference of less than 3,32 Ct between the positive and negative growth control) within 6 hours of incubation, it was excluded from analysis, since the difference between growth and inhibition was too small to reliably determine susceptibility or resistance.

### Rapid identification of blood culture isolates

For identification, a portion of the bacterial suspension in 0.9% saline, that was used for RAMAST, was centrifuged 16000× *g* for 5 minutes. The supernatant was carefully removed, after which the bacterial pellet was re-suspended in sterile demineralized water. Along with RAMAST, after Gram-staining, rapid identification of the strains was performed using a multiplex 16S DNA based PCR-assay described by Hansen et al [19]. By using genus- and species-specific probes, Gram-negative bacteria were divided into *Pseudomonas aeruginosa* and other Gram-negative species. Within the Gram-positive species, probes were used to identify *Staphylococcus* spp, *S.aureus*, *Enterococcus* spp and *Streptococcus* spp.

### Routine methods for identification and antibiotic susceptibility testing

Routine identification of GNRs was performed by the BD Phoenix Automated Microbiology System (BD Diagnostic Systems), simultaneously with antibiotic susceptibility testing. For the identification of *Staphylococcus* species, catalase-positive strains were tested for coagulase and DNAse production. If both tests were positive, the strain was identified as *S. aureus*. *Enterococcus* species were identified using bile esculin and tellur diagnostic tablets (Product no. 40411 and 45041 resp.; Rosco Diagnostica, Taastrup, Denmark) were used, according to manufacturer's guidelines. If both tests were positive, the strain was identified as *Enterococcus faecalis*, whereas in case of a positive bile esculin test but a negative tellur test, an API 20 Strep test (Biomérieux SA, Marcy l'Etoile, France) was performed to further identify the strain.

For routine antibiotic susceptibility testing, the Phoenix system was used, except or *Pseudomonas aeruginosa*, for which disk diffusion according to CLSI-guidelines was performed [17].

### Gold standard for antibiotic susceptibility testing

Results of antibiotic susceptibility testing with the new method were analyzed anonymously for categorical agreement with the results of microbroth dilution according to CLSI-guidelines, which was used as the gold standard [17]. Errors were defined as minor, major or very major. A major error represented a false resistant result, and a very major error was defined as a false susceptible result, as described previously [20]. When RAMAST showed a susceptible result where the gold standard showed an intermediate resistant result, this was a minor error.

## Results

### Phase I: Growth curves of bacteria from positive blood cultures

**Figure 1:**
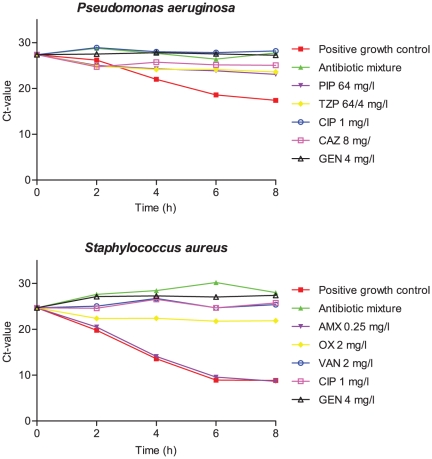
Growth kinetics of *P. aeruginosa* and *S. aureus* from blood cultures with and without antibiotics. AMX, amoxicillin; OXA, oxacillin; VAN, vancomycin; CIP, ciprofloxacin; GEN, gentamicin; AMC, amoxicillin/clavulanic acid; PIP, piperacillin; TZP, piperacillin/tazobactam; CAZ, ceftazidim.

First, real-time PCR was used to determine the kinetics of growth and the optimal incubation time for Gram-positive and Gram-negative bacteria, with and without antibiotics. Two representative examples of growth curves of Gram-positive cocci and Gram-negative rods established by RAMAST are shown in Figure 1. The growth curves showed that blood culture isolates require 6 hours of incubation before sufficient growth has occurred for reliable antibiotic susceptibility testing with RAMAST.

### Phase II: Susceptibility testing on positive blood cultures with RAMAST

A total of 114 blood cultures met the inclusion criteria and were tested for antibiotic susceptibility with the new method for RApid Molecular Antibiotic Susceptibility Testing (RAMAST): 85 GNRs, 19 *S. aureus* and 10 *Enterococcus* spp. (table 2). Two blood cultures had to be excluded due to insufficient growth within the 6 hours incubation period. These were one *Stenotrophomonas maltophilia* strain and one non-fermenting strain which could not be further identified. In total, 836 antibiotic-isolate combinations were tested.

**Table 2:** Tested isolates.

Species	N
**Gram-positive cocci**	**29**
*Staphylococcus aureus*	19
*Enterococcus faecium*	7
*Enterococcus faecalis*	3
**Gram-negative rods**	**85**
*Escherichia coli*	49
*Pseudomonas aeruginosa*	11
*Enterobacter cloacae*	7
*Klebsiella pneumoniae* species *pneumoniae*	7
*Klebsiella oxytoca*	2
*Proteus mirabilis*	2
*Serratia marcescens*	1
*Proteus vulgaris*	1
*Klebsiella* species	1
*Salmonella typhimurium*	1
*Morganella morganii*	1
*Aeromonas hydrophila*	1

Categorical agreement of RAMAST for *S. aureus* was 97.9%. Both errors in this group occurred with amoxicillin. All other tested antibiotics showed an agreement of 100% (table 3). In the group of *Enterococcus* spp, one major error was found, resulting in an agreement of 95%.

**Table 3:** Agreement and errors in the tested population, per antibiotic.

Antibiotic	% Susceptible	% Agreement	No. minor errors	No. major errors	No. very major errors
*Staphylococcus aureus* (N = 19)					
Amoxicillin	36.8%	89.5%	0	0	2
Gentamicin	100%	100%	0	0	0
Oxacillin	100%	100%	0	0	0
Trimethoprim/sulfamethoxazole	100%	100%	0	0	0
Vancomycin	100%	100%	0	0	0
*Enterococcus* spp (N = 10)					
Amoxicillin	30%	95.0%	0	1	0
Vancomycin	100%	100%	0	0	0
Gram-negative rods (N = 85)					
Amoxicillin	40.0%	97.3%	0	2	0
Amoxicillin/clavulanate	70.7%	93.3%	4	0	1
Ceftazidime	91.9%	98.8%	0	1	1
Ciprofloxacin	89.4%	98.8%	1	0	0
Gentamicin	94.1%	100%	0	0	0
Piperacillin	87.1%	87.1%	9	0	2
Piperacillin/tazobactam	97.3%	100%	0	0	0
Trimethoprim/sulfamethoxazole	71.8%	96.5%	0	3	0

Gram-negative rods showed an agreement of 96.7%, with a minor error rate of 1.9%, a major error rate of 0.8% and a very major error rate of 0.6%. The majority of errors in this group (n = 11) occurred with piperacillin and all but one of these errors occurred in *Escherichia coli* strains, one error occurred with an *Enterobacter cloacae* strain. All other antibiotics showed an agreement of >93% (table 3).

In this study, the routine methods (Phoenix system and disk diffusion) were shown to have an agreement with the gold standard of 96.8% for *S.aureus*, 95.0% for *Enterococcus* spp. and 97.4% for GNRs.

## Discussion

Here we describe a new method, RAMAST, for antibiotic susceptibility testing directly on positive blood cultures combining culture and real-time PCR. An overall agreement of ≥95% was shown for *S.aureus*, *Enterococcus* spp. and GNRs.

For *S.aureus*, all antibiotics showed a 100% agreement, except for two errors for amoxicillin. This is comparable with the results of the Phoenix system, which also showed errors for amoxicillin in *S.aureus* in this study.

In Gram-negative rods, the majority of antibiotics showed an agreement of ≥93%. Only piperacillin showed a high percentage of errors, which was also found in other methods for antibiotic susceptibility testing [21], [22], [23]. All but one of these errors occurred in amoxicillin-resistant *E.coli* strains, for which piperacillin would never be considered an appropriate treatment, so in clinical practice, these errors would not result in an inappropriate treatment. Nevertheless, although piperacillin would thus not be appropriate treatment of amoxicillin resistant E. coli, RAMAST results for piperacillin should not be reported in clinical practice.

The overall agreement of RAMAST with results of microbroth dilution was comparable to the agreement we found for the results of routinely used methods compared to microbroth dilution, which in this study was 96.8% for *S.aureus*, 95.0% for *Enterococcus* spp. and 97.4% for GNRs. The results of RAMAST also meet the criteria for selecting an antibiotic susceptibility system proposed by Jorgensen et al [20].

RAMAST is based on a method published by Rolain et al. [16], which combined culture and PCR for antibiotic susceptibility testing for a selection of ATCC reference strains. However, the method described by Rolain et al. was not useful for rapid antibiotic susceptibility testing on positive blood cultures. It used bacteria from agar, thus requiring an overnight subculture of the blood culture. Instead we used SSTs to harvest bacteria directly from positive blood cultures, which was described previously in other systems for antibiotic susceptibility testing [24], [25], [26], [27], [28], [29]. We found that these bacteria require 6 hours of incubation time for reliable antibiotic susceptibility testing. Rolain et al., who used bacteria from a fresh culture of reference strains on agar, required only 2–4 hours of incubation time. Bacteria from positive blood cultures may be in a stationary state because nutrients in the blood culture broth are depleted due to the high bacterial load [30]. This might explain why bacteria harvested directly from positive blood cultures require more incubation time for sufficient growth.

In contrast to Rolain et al., the PCR-assay that was used for RAMAST did not require an extensive DNA-isolation [18]. Instead, washing the incubated diluted blood culture once was sufficient, saving time and money. This method was also used for the identification PCR-assay and may also prove useful for other PCR-assays on positive blood cultures. Another advantage of this universal 16S rRNA PCR-assay is that, in contrast to the genus- or species specific PCR-assays used by Rolain et al., it can be used for all bacteria, which further simplified the method.

Time required for antibiotic susceptibility testing using RAMAST could therefore be reduced to only 9 hours, starting from a positive blood culture.

Turnaround time of many systems for antibiotic susceptibility testing, like broth microdilution [24], [25], or automated systems like the BD Phoenix system [26] or Vitek 2 [27], [28] can also be reduced with one day by the use of SSTs. Many of these direct methods however showed disappointing results for Gram-positive cocci (GPCs) [28], [31] or they were not tested at all [26], [32]. Our method can be applied to the majority of clinically relevant GPCs and (facultative) aerobic GNRs. It allows for accurate antibiotic susceptibility testing within 9 hours, which for most strains is more rapid than with these direct methods. This makes the test especially useful for laboratories with extended opening hours or 24-hour laboratories.

Many studies have shown that starting early with appropriate empirical therapy leads to a better prognosis for the patient [7], [8], [9]. More rapid identification and antibiotic susceptibility testing on positive blood cultures can reduce the time that inadequate antibiotic therapy is administered [13], [14], [15], [33], [34]. In this study RAMAST was combined with the rapid identification method described by Hansen et al [19], using a multiplex PCR-assay which required only 3 hours to perform. Alternatively, other rapid methods for identification can be used in combination with RAMAST; for example matrix-assisted laser desorption ionization time-of-flight (MALDI-TOF) mass spectrometry [35] or LightCycler® SeptiFast Test MGRADE (Roche).

In conclusion, our study shows that RAMAST can potentially provide accurate results for antibiotic susceptibility testing for the majority of clinically relevant blood culture isolates. Since the procedure can be applied directly on positive blood cultures and can be completed within 9 hours, results are available in less time than other available methods for antibiotic susceptibility testing.
